# Social wellbeing profiles: associations with trust in managers and colleagues, job satisfaction, and intention to leave

**DOI:** 10.3389/fpsyg.2024.1157847

**Published:** 2024-06-24

**Authors:** Eugeny C. Hennicks, Marita M. Heyns, Sebastiaan Rothmann

**Affiliations:** Optentia Research Unit, North-West University, Vanderbijlpark, South Africa

**Keywords:** social wellbeing, trust, job satisfaction, intention to leave, latent profile analysis

## Abstract

**Introduction:**

This study aimed to determine latent social wellbeing profiles and investigate differences between the profiles in terms of trust in managers and colleagues, job satisfaction, intention to leave, and demographic variables (age and service years).

**Methods:**

Permanently employed individuals of a South African utility organization participated in the study (*N* = 403). The Social Well-being Scale, Workplace Trust Survey, Job Satisfaction Scale, and Turnover Intention Scale were administered.

**Results:**

Four social wellbeing profiles were identified: socially disconnected (19.11%), socially challenged (31.27%), socially adequate (22.30%), and socially thriving (27.33%). Individuals in the socially thriving profile were significantly more inclined to experience job satisfaction and had lower intentions to leave than individuals in the other social wellbeing profiles. Two demographic variables, namely, age and service years, were associated with profile membership.

**Discussion:**

This study provided a nuanced understanding of social wellbeing by identifying patterns in which social contribution, social integration, social actualization, social coherence, and social acceptance interacted within individuals in a population, which might otherwise not have been evident. The differing levels of social wellbeing among these profiles have substantial implications for job satisfaction and staff retention.

## 1 Introduction

People’s sense of belonging, acceptance, and meaningful contribution is greatly influenced by their sense of social wellbeing in organizations (Lovejoy et al., 2021; Santos and Lousã, 2022). In addition to reducing isolation, social wellbeing fosters positive social interactions and is associated with greater life satisfaction, resilience, and health (Weziak-Bialowolska et al., 2022). Prioritizing social wellbeing is crucial for creating inclusive and thriving organizations and communities in an era of social fragmentation and technological isolation (Singh and Gautam, 2023). Waldinger and Schulz (2023) found that the quality of relationships is the most critical factor influencing employees’ wellbeing. Because the social arrangements in societies have failed to keep up with economic, political, technological, and cultural changes, individuals are deprived of the social lives they need to survive. According to Cohen (2022), most managers underestimate the degree to which employees are motivated by the need to belong and to feel connected to others at work. Consequently, feelings of loss and anomie become overwhelming.

The importance of social wellbeing at work is evident from Clifton and Harter’s (2021) findings that having a best friend at work makes employees more productive and effective. However, only 3 out of 10 employees reported having a best friend. Organizations that double the percentage of their employees who have a best friend at work will be able to increase their customer ratings, reduce safety incidents, and increase profits by 10%. In addition, chronic loneliness and social isolation are detrimental to an individual’s physical and mental health (Clifton and Harter, 2021). Courtin and Knapp (2017) found that the social isolation of employees adversely impacts individual and organizational outcomes.

According to Colenberg et al. (2021), many studies have focused on employee wellbeing’s physical and emotional dimensions. Although fewer studies focused specifically on social wellbeing in the workplace, various frameworks have been developed, highlighting its multifaceted nature and significance. These frameworks explored organizational identification (Mael and Ashforth, 1992), in-group identification (Leach et al., 2008), relational identity and social support networks (Sluss et al., 2012), psychological safety (Edmondson, 2019, 2023), loneliness (Wright et al., 2006), and positive relationships (Dutton and Ragins, 2017). These theories and models provide valuable insights into developing supportive, inclusive, and engaging work environments and contribute to individual and organizational success.

Keyes (1998, 2024) contends that social life provides a foundation for defining social wellbeing based on its benefits through individuals’ appraisals of their circumstances and functioning institutions in terms of five challenges: social integration (sharing values and goals with others and feeling part of a larger social structure); social acceptance (perceiving others as generally accepting and positive in the organization); social contribution (believing one is an important member of an institution, having something valuable to contribute); social actualization (having a hopeful view of the institution and recognizing its potential); and social coherence (finding the social world to be comprehensible and meaningful).

Addressing social wellbeing in the workplace is crucial in South Africa due to the broader socio-economic challenges, including inequality and unemployment. The organization can promote inclusivity and equity by prioritizing social wellbeing. Moreover, focusing research on the social wellbeing, trust, job satisfaction, and retention of managers in the utility industry in South Africa is vital for enhancing the organization’s effectiveness, stability, and reputation. As a public utility, the organization is responsible for providing reliable services to the community. A satisfied and well-supported workforce will likely deliver high-quality service, enhancing the utility’s reputation and public trust. Understanding the associations between trust in managers and colleagues and social wellbeing profiles is essential for the flourishing of individuals (Keyes, 2024). Moreover, scientific information about the associations between managers’ social wellbeing profiles, job satisfaction, and intentions to leave is needed.

Social wellbeing can be studied using variable- and person-oriented approaches. Variable-oriented analyses examine the average relationships among variables in specific samples (Meyer and Morin, 2016; Caesens et al., 2020; Wang and Wang, 2020). The assumption of heterogeneous populations within subgroups of samples is fundamental to latent profile analysis (LPA; Caesens et al., 2020).

Quantitative effects are easily handled in continuous latent variable models (e.g., regression analyses). However, qualitatively different profiles (obtained through LPA) are a valuable alternative for providing new and theoretically interesting information (Spurk et al., 2020). LPA assumes that people can be classified according to the closeness of relationships between the dimensions with varying degrees of probability with different profiles. The approach makes it possible to identify social wellbeing profiles for diverse employees in an organization (Meyer and Morin, 2016). Previous studies investigating the effects of social wellbeing have utilized factor analysis to examine the association between social wellbeing, job satisfaction, and intention to leave (see Hennicks et al., 2022). However, no studies have focused on different social wellbeing (as conceptualized by Keyes, 1998) profiles in relation to their covariates and outcomes. Therefore, a research gap was evident based on limited knowledge about social wellbeing and its impact on organizational outcomes.

## 2 Social wellbeing

### 2.1 Dimensions of social wellbeing

Keyes (1998) developed the most prominent conceptualization of social wellbeing (Colenberg et al., 2021). Keyes (1998) defines social wellbeing as a combination of five distinctive dimensions: social integration, social acceptance, social coherence, social contribution, and social actualization.

In work contexts, social integration refers to evaluating the quality of one’s relationship with an organization. Integration consists of people feeling a sense of belonging to their organization and having something in common with the individuals who constitute their social reality or work community. People function better when they feel like they belong to an organization (Cohen, 2022). However, research has shown that most people underestimate the degree to which people are driven by the need to belong (Heath, 1999; Cohen, 2022). According to Keyes (1998), social integration draws on conceptions of social cohesion and shared norms (Durkheim, 1951) as opposed to cultural estrangement and social isolation (Seeman, 1991). Through social coordination, individuals can express their affinity for society and connection to one another (Durkheim, 1951). Conversely, the absence of meaningful, supportive relationships leads to social isolation (Seeman, 1991) and belongingness uncertainty (Walton and Cohen, 2007), a state of mind characterized by individuals’ doubts about whether others accept them in a work context (Cohen, 2022).

Social contribution entails employees’ appraisals of their social value to their organization (Keyes, 1998; Rothmann, 2013). The belief that one is a vital member of the organization, that one is respected and that one’s daily work tasks add value to one’s department or work team is an aspect of social contribution (Redelinghuys et al., 2019). In addition, social contribution indicates whether and to what extent people feel that what they do is valued by their organization and contributes to the common good (Prilleltensky and Prilleltensky, 2021). Social acceptance refers to the evaluation of one’s social value. It indicates favorable views of human nature, tolerance for individual differences, and positive attitudes toward colleagues. Individuals who exhibit social acceptance trust others, believe they can be kind, and think they can be industrious. Socially accepting individuals have a positive view of human nature, are tolerant of others, and are comfortable around them (Keyes, 1998; Colenberg et al., 2021).

Social actualization concerns the evaluation of the potential and the trajectory of the organization. It entails perceiving that the organization is growing and developing, which realizes its members’ social potential (Keyes, 1998; Rothmann, 2013). It reflects employees’ hopefulness about the condition and future of the organization, including optimism that its potential can become a reality through the collective contribution of its members (Keyes and Shapiro, 2004). Individuals who are socially well can envision that they, and people like them, are potential beneficiaries of social growth.

Social coherence refers to the perception of quality, organization, and operation of social phenomena, including their social support and network structures (Keyes, 1998; Rothmann, 2013). Individuals with social coherence feel that their society is sensible and predictable. They feel they can understand what is happening around them and find meaning in how the organization functions (Keyes and Shapiro, 2004; Colenberg et al., 2021). When people are socially well, they care about the kind of world they live in – moreover, individuals with coherence attempt to protect and maintain coherence when faced with organizational turbulence and uncertainty.

### 2.2 Latent profiles of social wellbeing

We contest the likelihood of homogeneous social wellbeing profiles because workforces across the globe have become increasingly diverse and may include individuals from various cultures with diverging value orientations (Tuzun and Kalemchi, 2012). For example, conceptualizations of the self, wellbeing, and the importance of social relationships in Asian and African countries seem to diverge from assumptions in Western contexts (Wissing et al., 2022). Individuals from collectivist cultures define themselves by group membership. Consequently, they prioritize family and group goals over personal goals and are partial to close and long-term relationships. Individualistic cultures, in contrast, place a high premium on personal goals and self-actualization over those of groups. Collectivist and individualistic cultures differ in their stance toward power distance in relationships, the need for harmonious relations with equals, and the importance of individual progress. These differences find expression in how employees relate to their colleagues, team members, leaders, and the organization and may differentially influence work-related attitudes, behavior, and work outcomes (Tuzun and Kalemchi, 2012).

While sensitivity to observed differences between individualistic and collectivist cultures can add a more inclusive and balanced understanding of social wellbeing, it cannot be assumed that people from the same culture are homogeneous. Research shows that there is substantial variation among individuals from the same cultural background; furthermore, individuals, regardless of cultural affiliation, consider collectivist and individualistic perspectives to varying degrees to selectively shape their preferences, which are, in part, also determined by the situation or context in which they find themselves (Tuzun and Kalemchi, 2012; Wissing et al., 2022). In addition, social stigma affects social identity and one’s desire to associate with a particular group (Zhang et al., 2020). Social stigma-based rejection experiences promote social isolation and extend impaired trust relations and social functioning beyond the reaction to a specific offender or context of abuse (Zhang et al., 2020). In contrast, individuals who sense camaraderie with their fellow workers and who experience connectedness to the organization as an entity tend to be more trusting, are more open to the opinions of others, and are more likely to experience a sense of community, belonging, and pride; they are also more likely than those who do not share similar experiences to feel that they can contribute to organizations in meaningful ways (Steffens et al., 2017).

Previous research has confirmed an association between social identification with work groups and an individual’s wellbeing; however, the effect of more proximal groups on social wellbeing is poorly understood (Steffens et al., 2017). Individuals do not necessarily function in a single work-related entity but may share multiple group memberships. Their social experiences within these groups may vary, resulting in a situation where they may relate more strongly to some groups than others and experience varying degrees of social acceptance and belonging in different groups. Given these findings, it seemed important to guard against a one-sided, simplistic approach that assumed that all people valued different aspects of social wellbeing similarly or that their experiences in distant and more proximal groups had a similar effect on their social wellbeing.

The current study contributes to the literature by uncovering the patterns that characterized the combination of different social wellbeing dimensions and their effects, which had thus far been unknown. No studies that explicitly focused on identifying employee social wellbeing profiles (as conceptualized by Keyes, 1998) in relation to their antecedents and outcomes in workplace contexts could be identified. However, some studies either employed a person-oriented approach to study the effects of social isolation in unrelated work contexts or employed dissimilar or multidimensional models of wellbeing.

A cross-national study using LPA examined social isolation’s effect on employees’ subjective wellbeing in the United Kingdom and France during the COVID-19-induced lockdown. The authors identified five distinct wellbeing profiles, showing that employees’ access to social support and the maintenance of strong social networks were differentially associated with resilience, coping with stress, and several other health-related benefits (Harju et al., 2021). In another study, Moller and Rothmann (2019) employed a multidimensional model of flourishing at work and identified four mental health profiles for managers in a South African agribusiness. Their findings showed that managers less frequently experienced social wellbeing (compared to emotional and psychological wellbeing) in all profiles.

### 2.3 Trust, job satisfaction, intention to leave, and social wellbeing

The development of societal norms affects the formation of social relations. This formation depends on trust, which fertilizes relationships and is more inclined to develop among workers who share social identities (Helliwell and Wang, 2011); trust is needed to build a thriving organization. Thus, trust is a crucial component of governing social relations and social exchange, as high-trusting environments produce more resilient employees (Schoorman et al., 2007; Helliwell et al., 2016); however, protests, civil unrest, corruption, and retrenchments increase skepticism, weigh down trust deficits, and erode social relations (Crosby, 2016). Workplace trust relationships can play an important role in facilitating employee wellbeing, since previous research has acknowledged that trust drives the development of effective relations and fundamentally influences organizational social environments (Hennicks et al., 2022). Research has also shown that trust regulates an employee’s attitude toward developing social relations, drives further trust, and strengthens social ties (Koo et al., 2019; Karatepe et al., 2020).

Furthermore, trust has become increasingly critical as organizations face increasing uncertainty brought on by corruption, political unrest, retrenchments, and questionable decision-making (Bachmann and Zaheer, 2006; Kramer and Cook, 2006; Cook et al., 2009; Hennicks et al., 2022). The magnitude of the impact of trust on wellbeing has been undermined, as the strength of social ties relies on trust (Gambetta and Hamill, 2005; Edelman and Associates, 2009). Therefore, trust violation distorts social equilibrium, harming work relationships (Ren and Gray, 2009).

Restoring and enhancing trust in organizations rely on creating a conducive work environment that stimulates the development of positive relations among workers. Building trust starts as early as integrating new employees into an organization; here, social actualization is key, as feelings of identification and belonging fuel the need to build relations with colleagues through the socialization process, and improved trust starts developing (Williams, 2001; Allen and Rhoades Shanock, 2013). The level of trust in colleagues differs from that in managers based on hierarchical power (Schoorman et al., 2007). Employees may be more risk-averse regarding trusting those who influence them, whereas a different set of considerations influences the development of trust among colleagues (Schoorman et al., 2007).

Trust between colleagues is critical, as it facilitates social integration through psychological safety and contributes to daily operational functioning because employees support and depend on one another for the attainment of set organizational goals and have faith in what is promised versus actions (Ferres et al., 2004; May et al., 2004; Ho and Astakhova, 2018). These factors yield not financial benefits but greater social rewards beneficial to employee functioning and wellbeing (Van Dyne et al., 1994). Individuals who suspect that others are prejudiced against them or feel isolated may mistrust their colleagues, which, in turn, may affect their social identification with the group (Zhang et al., 2020). However, they may still identify with the mission and values of the organization.

Trust in managers is equally important, as it allows employees to open up and share sensitive information crucial for business survival. It also allows employees to openly express their feelings about their work situation, which leads to assurance and increased trust (Hassan and Ahmed, 2011). Hsieh and Wang (2015) confirmed that trust in managers was linked to positive organizational outcomes. Employees need to return increases in authentic behavior, which can provide a safe space for trust to develop.

Social relationships with managers and colleagues have been shown to “pay off” in positive work outcomes. These outcomes can be leveraged further by incorporating the role of trust within the workspace; this highlights the importance of social exchange, rooted in social exchange theory (Blau, 1964), which states that employees reciprocate what they receive. However, despite clear-cut benefits stemming from trust, research has indicated that more research is needed to understand social wellbeing and its association with trusting relations. Employees who experience poor social relations may languish by feeling “stuck” and obligated to be in a relationship with superiors. In contrast, good relations with colleagues and managers build trust, which signifies that employees have good connections with individuals in their work organization (Karatepe, 2012).

Previous research has conceptualized trust by focusing extensively on horizontal trust relationships such as trust in colleagues. Much of the trust literature has failed to examine trust from a social wellbeing perspective. The scarcity of literature addressing associations between workplace trust relationships and social wellbeing indicated that this study was of great importance, as the magnitude of the link between social wellbeing and its association with trust in supervisors and colleagues needs to be established.

Employees residing in a positive, supportive workspace trust their organization and develop an emotional attachment and a sense of commitment, evident through increased job satisfaction (Karatepe, 2012; Ho and Astakhova, 2018). Employees who experience this feel indebted to the organization and are less likely to exit because they tend to align plans and career goals. They fear losing out on non-monetary benefits such as social support structures, training, and development (Ampofo, 2020).

Positive workplace relations boost employees’ social wellbeing, promoting in-role and innovative job performance (Khoreva and Wechtler, 2018); unsatisfactory relationships with colleagues and managers affect employees’ intention to leave an organization (Janik and Rothmann, 2015). Intention to leave, signifying a deliberate wish to leave the organization in the foreseeable future, is the final stage of a withdrawal cognition process that accurately predicts actual turnover, even more so than affective indicators such as job satisfaction (Tuzun and Kalemchi, 2012).

A study by Caesens et al. (2020) supported the notion that employees who experienced high levels of social support from the organization, their managers, and their colleagues were more inclined to feel obligated to reciprocate with positive work attitudes and behaviors. In contrast, socially isolated people were associated with more undesirable work attitudes and behaviors, such as lower levels of job satisfaction, performance, and commitment and higher levels of emotional exhaustion and absenteeism. Furthermore, a longitudinal LPA study by Caesens et al. (2021) confirmed that different sources of support – whether the support was received from the organization, a manager, or a colleague – might all have distinct effects on employees’ wellbeing and their functioning and that these associations appeared to be consistent over time.

Employees of different ages and service years might differ in their levels of social wellbeing. Younger employees might value social integration, as being accepted by an already established social group might be difficult. Social values are embedded in societal norms, which influence employee cognitive and social structures within a group residing in a society, a team, or an organization (Peterson and Barreto, 2014). Caesens et al. (2021) acknowledged research by Ng and Feldman (2010) that linked tenure and age to lower levels of psychological difficulty but did not find these aspects to be significant predictors of profile membership in their study. De Jager et al. (2014) did not find indications of significant differences between overall social wellbeing across various age groups. However, they found significant differences between employees older than 50 (compared to those in the 20 to 30 age group) regarding their sense of belonging and social value.

### 2.4 Current study

This study focused on the social wellbeing of individuals employed by a significant utility organization in South Africa. Given the diversity of the organization’s staff, this study challenged the individual homogeneity of social wellbeing. The present study aimed to uncover whether age and service years are associated with social wellbeing profiles. We also aimed to uncover how individuals in different social wellbeing profiles differ regarding trust in their managers and colleagues and two work outcomes: job satisfaction and intention to leave.

By estimating social wellbeing profiles, we facilitated a more in-depth understanding of this construct and how it was configured among individuals in the utility organization. Moreover, we assessed the association between two different foci of trust, selected demographic variables, and social wellbeing profile membership. In short, the originality of this research stemmed from methodically identifying and comparing specific subgroups of employees in terms of their social wellbeing configurations and how these patterns related to trust relationships at work and complexities as influenced by a demographically diverse workforce composition.

## 3 Materials and methods

### 3.1 Participants

Consenting participants (*N* = 403) from various designation levels who were permanently employed in the utility industry in South Africa across various provinces responded by fully completing an online questionnaire. Employees who contributed to the study had a minimum qualification level of Grade 12. A postgraduate qualification ranked as the highest qualification since 51.6% of participants had obtained that level of education and occupied low, middle, or senior positions in the industry. Most employees had dedicated a minimum of 11 years of service, on average, to the organization.

### 3.2 Measuring instruments

A biographical questionnaire was developed by the researcher and was aimed at measuring demographics.

The *Social Well-being Scale* (SWBS; Keyes, 1998), as adapted for a South African workplace context by Rautenbach (2015) and further extended by Redelinghuys (2016), was used to assess social wellbeing in terms of five subdimensions: social integration (three items, e.g., “During the past month at work, how often did you feel that you really belong to your organization?”); social acceptance (three items, e.g., “During the past month, how often did you feel that people in your organization are basically good?”); social contribution (three items, e.g., “During the past month, how often did you feel that you had something important to contribute towards your organization?”); social actualization (three items, e.g., “During the past month at work, how often did you feel that your organization is becoming a better place for people like you?”); and social coherence (three items, e.g., “During the past month, how often did you feel that the way your organization works, makes sense to you?”). The items were scored on a six-point scale, ranging from 1 (*never*) to 6 (*every day*). Respondents had to answer questions regarding the frequency with which they had experienced specific symptoms of social wellbeing during the past month.

Two subscales of the *Workplace Trust Survey* (WTS; Ferres, 2003; Ferres et al., 2004; Lehmann-Willenbrock and Kauffeld, 2010), namely, trust in managers and trust in colleagues, were used for this study. The WTS consisted of two subscales, namely trust in managers (nine items, e.g., “I feel that my supervisor keeps personal discussions confidential”) and trust in colleagues (12 items, e.g., “I feel I can trust my co-workers to do their jobs well”). The response options ranged from 1 (*strongly disagree*) to 7 (*strongly agree*). Correlations between the WTS and known instruments were significant, confirming the survey’s concurrent validity (Lehmann-Willenbrock and Kauffeld, 2010). As for convergent validity, Kleynhans et al. (2021, 2022) observed positive associations between WTS factors, authentic leadership, and flourishing.

The *Job Satisfaction Scale* (JSS; Saks, 2006) was used to measure job satisfaction. Five items measured how satisfied individuals were with their jobs (e.g., “Most days I am enthusiastic about my work” and “I consider my job rather unpleasant”). Response options ranged from 1 (*totally disagree*) to 5 (*totally agree*).

The *Turnover Intention Scale* (TIS; Sjöberg and Sverke, 2000), used to measure intention to leave, comprised three items (e.g., “I am actively looking for other jobs”), with response options ranging from 1 (*strongly disagree*) to 5 (*strongly agree*).

### 3.3 Research procedure

The researcher obtained permission from the highest level of authority in the organization to conduct the study in the utility industry. The North-West University of South Africa granted ethics clearance (NWU-00745-20-A4), and all ethical standards as prescribed by relevant legislation were adhered to during the study. The questionnaire was presented in the form of an electronic booklet explaining the purpose of the study, emphasizing confidentiality, and specifying processes to follow in case further clarity was needed. Participants were informed that they could withdraw from the study at any time. An independent service provider gathered, monitored, and checked the data collection process. Only once consent had been granted via permission forms could participants access and participate in the online survey provided. Participants had to complete all items on a specific page before proceeding to the next question. This approach eliminated all the risks associated with instances of missing values. A period of 2 weeks was allocated to complete the online survey. The researchers accessed results using an anonymized format to analyze data accordingly.

### 3.4 Data analysis

The data was analyzed using SPSS 29.0 (IBM Corp, 2022) and Mplus 8.11 (Muthén and Muthén, 1998–2024; Wang and Wang, 2020). Confirmatory factor analysis (CFA) was utilized to test the measurement model, which consisted of trust in the manager and colleagues, social wellbeing subscales, and work outcomes (job satisfaction and intention to leave). The Maximum Likelihood with Robust Standard Errors (MLR) estimator in Mplus 8.11 was used. The MLR estimator is useful because it provides robust standard errors and chi-square test statistics less sensitive to non-normality. Fit indices such as the chi-square statistic, standardized root mean residual (SRMR), root mean square error of approximation (RMSEA), Tucker-Lewis index (TLI), and comparative fit index (CFI) were used to assess model fit. Relationships between the variables were identified by using Pearson correlation coefficients. Effect sizes ranged between small (*r* ≥ 0.10), medium (*r* ≥ 0.30), and large (*r* ≥ 0.50), keeping to the guidelines set by Cohen (2013). This study made use of omega coefficients (as opposed to alpha coefficients) as indicators of scale reliability (Wang and Wang, 2020). The cut-off value for scale reliability was set at 0.70 (Nunnally and Bernstein, 1994). Descriptive statistics were used to describe the data. In our investigation of the relationship between age and service years, Cramer’s V was calculated from the Chi-square statistic obtained from the contingency table of the two categorical variables. Cramer’s V is a robust, interpretable, and flexible measure ideally suited for analyzing the association between age categories and service years (Field, 2024).

Latent profile analyses were performed to identify groups within different categories that affected social wellbeing (Orpinas et al., 2015; Spurk et al., 2020). Simulation studies suggest a minimum sample size of 300–500 for an LPA study. However, estimating the sample size needed in LPA is currently impossible using a simple formula or calculator. LPA assumes that within-class outcomes are locally independent and normally distributed within each class (Ferguson et al., 2020). The number of latent groups is unknown priori in exploratory applications of LPA (Orpinas et al., 2015). Thus, this study compared models with increasing numbers of latent groups to find an appropriate model, resulting in the number of profiles that best fit the data. There was no single criterion that determined the best solution. Analyses of models were instead based on theory, interpretability, and statistical criteria (Marsh et al., 2009; Spurk et al., 2020). When choosing the model to fit these data, parsimony (i.e., allowing for data complexity with the fewest latent classes) was a priority (Orpinas et al., 2015).

The final profile solution should consider multiple fit values and content decision criteria, including error messages and out-of-bound parameters (Meyer and Morin, 2016; Spurk et al., 2020). The Bayesian information criterion (BIC), Akaike information criterion (AIC), sample-size adjusted BIC (ABIC), the Vuong-Lo-Mendell-Rubin likelihood ratio test, the Lo-Mendel-Rubin (LMR LR) test, the adjusted LMR LR (ALMR) test, and the bootstrapped likelihood ratio test (BLRT; Nylund et al., 2007), were used to compare the different profiles (Marsh et al., 2009; Wang and Wang, 2020). Effective use of LPA depends on considering average latent class probabilities for individuals assigned to each class (Geiser, 2013). Finally, entropy was used to determine the quality of the classification of the LPA, with values closer to 1 indicating a good classification of the profiles (Celeux and Soromenho, 1996; Pastor et al., 2007). Average latent class probabilities and entropy values above the recommended .80 threshold indicate high confidence in correctly classifying individuals into profiles (Spurk et al., 2020).

The AUXILIARY (BCH) function in Mplus 8.11 was used to compute the associations between profile membership, trust in managers and colleagues, job satisfaction, and intentions to leave (Asparouhov and Muthén, 2014; Bakk and Vermunt, 2016). Two demographic variables, namely age category and service years, were used to predict profile membership using multinomial logistic regression.

## 4 Results

### 4.1 Confirmatory factor analysis

A measurement model consisting of the five social wellbeing scales, namely social contribution (3 items), social integration (3 items), social actualization (3 items), social acceptance (3 items), and social coherence (3 items), the workplace trust scales (trust in managers, 9 items, and trust in colleagues, 12 items), job satisfaction (4 items), and intentions to leave (3 items) was tested using confirmatory factor analysis. The variables were allowed to correlate. The CFA showed acceptable fit: χ^2^ = 1,559.56 (df = 824), *p* < 0.0001; RMSEA = 0.05 [(0.04, 0.05), *p* = 0.912]; CFI = 0.93; TLI = 0.93; SRMR = 0.04. Inspecting the loadings and cross-loadings, the overall size of the factor loadings of the items on their target factors was acceptable (social contribution: λ = 0.69–0.82; mean = 0.77; social integration: λ = 0.72–0.78; mean = 0.76; social actualization: λ = 0.78–0.85; mean = 0.82; social acceptance: λ = 0.72–0.90; mean = 0.81; social coherence: λ = 0.82–0.85; mean = 0.83), trust in supervisor: λ = 0.77–0.93; mean = 0.89; trust in colleagues: λ = 0.75–0.89; mean = 0.83; job satisfaction: λ = −0.45 to 0.81; mean = 0.71; and intention to leave: λ = 0.75–0.94; mean = 0.85, showing well-defined factors corresponding to *a priori* expectations.

### 4.2 Descriptive statistics, reliabilities, and correlations

Table 1 reports the variables’ descriptive statistics (means and standard deviations), omega reliabilities, and Pearson correlations.

**TABLE 1:** Descriptive statistics, omega reliabilities, and Pearson correlations between the variables.

Item	Mean	SD	ω	1	2	3	4	5	6	7	8
Social contribution	4.60	1.40	0.81	–	–	–	–	–	–	–	–
Social integration	4.07	1.53	0.81	0.61**	–	–	–	–	–	–	–
Social actualization	3.03	1.53	0.86	0.47**	0.64**	–	–	–	–	–	–
Social acceptance	3.68	1.49	0.86	0.42**	0.57**	0.64**	–	–	–	–	–
Social coherence	3.58	1.60	0.87	0.47**	0.64**	0.72**	0.63**	–	–	–	–
Trust – manager	5.02	1.64	0.97	0.23**	0.43**	0.33**	0.37**	0.33**	–	–	–
Trust – colleagues	4.86	1.37	0.96	0.29**	0.47**	0.44**	0.52**	0.42**	0.61**	–	–
Job satisfaction	3.59	0.95	0.80	0.29**	0.40**	0.37**	0.34**	0.35**	0.45**	0.37**	–
Intention to leave	2.69	1.36	0.89	−0.10*	−0.30**	−0.31**	−0.19**	−0.18**	−0.28**	−0.21**	−0.59**

Scale for items 1–5 = 6; scale for items 6–7 = 7; scales for items 8–9 = 5.

*p < 0.05;

**p < 0.01.

Table 1 shows acceptable reliability coefficients higher than 0.70 (Nunnally and Bernstein, 1994) for all the scales. The correlations in Table 1 show that trust in managers is statistically significantly related to trust in colleagues (large effect). Trust in managers and colleagues is statistically significantly and positively related to job satisfaction (all medium effects), except for the correlation between social acceptance and trust in colleagues (large effect). Job satisfaction is statistically significantly and positively related to all the social wellbeing dimensions, trust in managers, and trust in colleagues (all medium effects). Intention to leave correlates statistically significantly and negatively with all social wellbeing dimensions. However, medium effect sizes are evident for social integration and actualization).

### 4.3 Latent profile analysis

Latent profile analyses were conducted on the responses of the 403 participants. Mplus 8.11 was used to analyze the responses to the SWBS dimensions. The results of six different models are reported in Table 2.

**TABLE 2:** Comparison of different LPA models.

Profile	AIC	BIC	ABIC	LMR LR test *p-*value	ALMR LR test *p-*value	BLRT *p*-value	Entropy	Smallest class proportion
1 Profile	6,734.35	6,774.34	6,742.61	n/a	n/a	n/a	–	–
2 Profiles	5,526.62	5,590.60	5,539.83	0.0000**	0.0001**	0.0000**	0.92	185
3 Profiles	5,019.38	5,107.36	5,037.55	0.0000**	0.0000**	0.0000**	0.91	119
4 Profiles	4,815.09	4,927.06	4,838.21	0.0236*	0.0256*	0.0000**	0.90	77
5 Profiles	4,709.18	4,845.14	4,737.25	0.3379	0.3448	0.0000**	0.88	49
6 Profiles	4,600.22	4,760.17	4,633.25	0.0363*	0.0388*	0.0000**	0.89	47

AIC, Akaike information criterion; BIC, Bayesian information criterion; ABIC, adjusted Bayesian information criterion; LMR LR, Lo-Mendell-Rubin test; ALMR LR, adjusted Lo-Mendell-Rubin test; BLRT, bootstrapped likelihood ratio test.

*p < 0.05;

**p < 0.01.

The results in Table 2 show that the two-profile solution has a better fit than the one-profile solution (ΔAIC = −1,207.73, ΔBIC = −1,183.74, and ΔABIC = −1,202.78). The three-profile solution is significantly better than the two-profile solution (ΔAIC = −507.24; ΔBIC = −483.24; ΔABIC = −502.28). Furthermore, the four-profile solution better fits the three-profile solution (ΔAIC = −204.29; ΔBIC = −180.3; ΔABIC = −199.34). However, the five-profile solution (ΔAIC = −105.91; ΔBIC = −81.92; ΔABIC = 100.96) and the six-profile solution (ΔAIC = −108.96; ΔBIC = −84.97; ΔABIC = −104.00) also significant improvements. For 5 and 6 profiles, Mplus 8.11 showed a warning message that 92 and 163 perturbed starting values did not converge, which indicates that we are extracting too many profiles. Therefore, we decided to choose the four-profile solution. The LMR LR, ALMR LR, and BLRT of profiles 2 (*p* < 0.0001), 3 (*p* < 0.0001), and 4 (*p* = 0.0236, *p* = 0.0256, and *p* < 0.0001) were statistically significant. The 4-profile solution fitted the data best. It replicated the best log-likelihood value (−2,379.55) several times using the default number of starting values. To verify whether a better log-likelihood can be obtained, a second run increased the number of random starting values 10 times and found the same best-replicated value 240 times.

**FIGURE 1:**
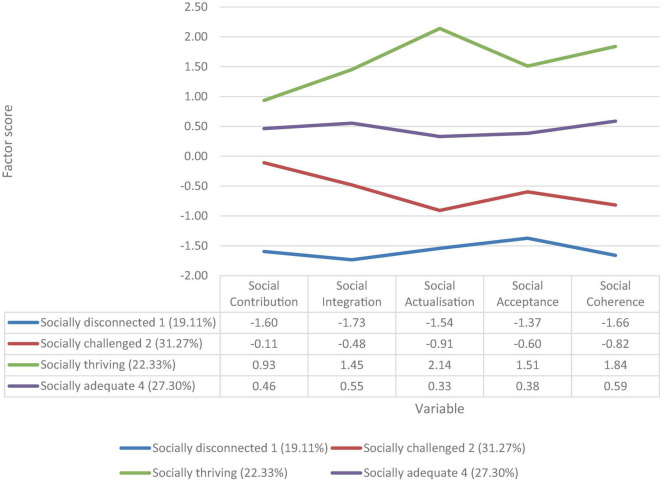
Latent profiles of social wellbeing.

The class proportions were acceptable for class 4. The entropy value of 0.90 indicated a highly acceptable classification. The average latent class probabilities, which are well above the cutoff value of 0.70 (Nagin, 2005), support correct profile assignment: Class 1 = 0.95; Class 2 = 0.94; Class 3 = 0.97; and Class 4 = 0.92 (Wang and Wang, 2020). The four classes are illustrated in Figure 1. The 4-profile model fits well, supporting the assumption of local independence.

A total of 77 people were assigned to Profile 1 (19.11%), 126 to Profile 2 (31.27%), 90 to Profile 3 (22.33%), and 110 (27.30%) to Profile 4. The size and sample proportion of the four profiles were not too small (Wang and Wang, 2020). The three latent profiles are illustrated in Figure 1.

Figure 1 and Table 3 show various tendencies. *Socially disconnected* individuals have low factor scores across the five social wellbeing dimensions (mean scores vary from −1.35 to 1.75), indicating significant struggles. They function poorly in terms of the following elements: the belief that their daily work tasks added value to their teams, departments, and organization; and their communal connectedness, valuing diversity, believing in others’ potential, and perceiving their relationships as coherent. *Socially challenged* individuals have mixed factor scores across the dimensions (means scores vary from −0.10 to −0.90). They scored the lowest on social actualization, acceptance, and coherence. Individuals in this profile do not have a positive view of humans and are uncomfortable around them. Their scores reflect that they are not hopeful about the condition and future of the organization and about being potential beneficiaries of social growth. Their understanding of what is happening in their organization is low.

**TABLE 3:** Descriptive statistics of the social wellbeing factor scores.

Variable	Disconnected		Challenged		Thriving		Adequate	
	Mean	SD	Mean	SD	Mean	SD	Mean	SD
Social contribution	−1.61	0.80	−0.10	0.74	0.93	0.36	0.48	0.59
Social integration	−1.75	0.45	−0.48	0.48	1.46	0.39	0.58	0.48
Social actualization	−1.54	0.43	−0.91	0.60	2.18	0.54	0.33	0.59
Social acceptance	−1.35	0.94	−0.61	0.94	1.53	0.55	0.40	0.73
Social coherence	−1.67	0.64	−0.82	0.71	1.86	0.43	0.59	0.67

*Socially thriving* individuals have high factor scores across all five dimensions (means scores vary from 0.93 to 2.18), indicating strong and positive social wellbeing. Individuals in this profile believe they are vital organizational members with something valuable to offer. They evaluated the quality of their relationship with the organization as high and experienced a sense of connectedness and belongingness. They have a positive view of humans, are comfortable around them, and believe their organization and social relations at work are meaningful and comprehensible. Most notably, individuals feel that the organization was evolving and could realize its potential. Finally, *socially adequate* individuals have positive moderate factor scores on the five dimensions (mean scores vary from 0.33 to 0.59), reflecting a moderate but not exceptional level of social wellbeing.

### 4.4 Associations between trust, social wellbeing profiles, job satisfaction, and intention to leave

Next, we examined the associations between the four social wellbeing profiles, trust in managers and colleagues, job satisfaction, and intentions to leave using the factor scores saved from the measurement model. This approach controls for measurement errors by giving more weight to items with smaller measurement errors (Wang and Wang, 2020). The automatic BCH approach for estimating the mean of a distal continuous outcome across latent profiles (Asparouhov and Muthén, 2014; Bakk and Vermunt, 2016) was used in this study. Table 4 shows the differences between the distal variables of the different social wellbeing profiles.

**TABLE 4:** Equality tests of means across profiles.

Trust: manager			Trust: colleagues		
	**Mean**	**SE**		**Mean**	**SE**
Disconnected	−1.18	0.22	Disconnected	−1.26	0.16
Challenged	−0.30	0.15	Challenged	−0.26	0.12
Thriving	0.90	0.12	Thriving	0.94	0.10
Adequate	0.90	0.12	Adequate	0.40	0.11
	**χ^2^**	***p***		**χ^2^**	***p***
Overall test	94.06	0.0000	Overall test	164.44	0.0000
Disconnected vs. thriving	71.31	0.0000	Disconnected vs. thriving	133.74	0.0000
Disconnected vs. challenged	10.60	0.0010	Disconnected vs. challenged	23.19	0.0000
Disconnected vs. adequate	37.97	0.0000	Disconnected vs. adequate	70.52	0.0000
Challenged vs. thriving	41.08	0.0000	Challenged vs. thriving	64.47	0.0000
Challenged vs. adequate	11.39	0.0010	Challenged vs. adequate	15.71	0.0000
Thriving vs. adequate	5.94	0.0150	Thriving vs. adequate	13.01	0.0000
**Job satisfaction**			**Intention to leave**		
	**Mean**	**SE**		**Mean**	**SE**
Disconnected	−0.83	0.11	Disconnected	0.32	0.13
Challenged	−0.23	0.08	Challenged	0.37	0.10
Thriving	0.72	0.07	Thriving	−0.57	0.10
Adequate	0.25	0.07	Adequate	−0.18	0.10
	**χ^2^**	***p***		**χ^2^**	***p***
Overall test	193.23	0.0000	Overall test	56.50	0.0000
Disconnected vs. thriving	155.34	0.0000	Disconnected vs. thriving	29.62	0.0000
Disconnected vs. challenged	18.73	0.0000	Disconnected vs. challenged	0.10	0.7470
Disconnected vs. adequate	69.99	0.0000	Disconnected vs. adequate	9.06	0.0030
Challenged vs. thriving	83.00	0.0000	Challenged vs. thriving	44.19	0.0000
Challenged vs. adequate	17.42	0.0000	Challenged vs. adequate	13.41	0.0000
Thriving vs. adequate	21.31	0.0000	Thriving vs. adequate	7.00	0.0080

*p < 0.01.

Table 4 shows that statistically significant differences exist between the social wellbeing profiles concerning trust in the manager (χ^2^ = 94.06, *p* < 0.0001) and colleagues (χ^2^ = 164.44, *p* < 0.0001). Concerning the differences between specific profiles, Table 4 shows that individuals in the socially thriving profile are statistically significantly more inclined to trust their managers and colleagues than those in the socially disconnected, socially challenged, and socially adequate profile. Individuals in the socially disconnected profile (compared to the challenged and adequate profiles) are statistically significantly less inclined to trust their managers and colleagues. Also, individuals in the adequate profile (compared to the challenged profile) are statistically significantly more inclined to trust their managers and colleagues.

Table 4 shows that statistically significant differences exist between the social wellbeing profiles regarding job satisfaction (χ^2^ = 193.23, *p* < 0.0001) and intentions to leave (χ^2^ = 56.50, *p* < 0.0001). Regarding the differences between specific profiles, Table 4 shows that socially thriving people are statistically significantly more inclined to experience job satisfaction and have lower intentions to leave than individuals in socially disconnected, socially challenged, and socially adequate profiles. Individuals in the socially disconnected profile (compared to those in the challenged and adequate profiles) are statistically significantly less satisfied with their jobs and have higher intentions to leave. Also, individuals in the socially adequate profile (compared to those in the challenged profile) are statistically significantly more satisfied with their jobs and have lower intentions to leave. However, the intention of employees in the socially disconnected profile to leave did not differ statistically significantly from the intention of the socially challenged profile (*p* = 0.7470).

### 4.5 Differences between demographic groups

Next, a multinomial logit model (Wang and Wang, 2020) was specified using Mplus 8.11 (Muthén and Muthén, 1998–2024). Age and service year categories were used to predict profile membership given the unique human resource profile of the specific public utility. The following age categories were created: 20–30 years (*n* = 27), 31–40 (*n* = 156), 41–50 (*n* = 117), 51 and older (*n* = 100). Furthermore, the following service year categories were created: 0–10 years (*n* = 135), 11–20 (*n* = 150), 21–30 (*n* = 79), and 31 years and more (*n* = 36). Cramer’s V examines the association between two categorical variables when there is more than a 2 × 2 contingency table. The Cramer’s V statistic was 0.48 (*p* < 0.001, medium effect).

Age (estimate = −0.63, SE = 0.30, *p* = 0.033) had a statistically significant negative effect in the multinomial model; younger individuals were more likely to be classified in the socially disconnected profile than the socially thriving one. Service years (estimate = 0.93, SE = 0.28, *p* = 0.001) had a statistically significant positive effect in the multinomial model; individuals with more service years were more likely to be classified in the socially disconnected profile than the socially thriving one. Also, individuals with more service years were more likely to be classified in the socially challenged profile than the socially thriving one (estimate = 0.59, SE = 0.23, *p* = 0.010).

## 5 Discussion

This study aimed to determine latent social wellbeing profiles and investigate differences between social wellbeing profiles in terms of trust in managers and colleagues, demographic variables (age and service years), and two work outcomes: job satisfaction and intention to leave. It was possible to replicate a measure of social wellbeing consisting of five distinguishable dimensions, as Keyes (1998) proposed. Four distinct social wellbeing profiles were evident in a large South African utility organization: socially disconnected, socially challenged, socially thriving, and socially adequate. These profiles were associated with trust in managers and colleagues, with significant differences between the social thriving profile and the other three. The varying levels of social wellbeing in the profiles have significant implications for staff job satisfaction and retention.

The socially disconnected profile was characterized by low functioning in all five dimensions of social wellbeing. Notably, employees within this profile exhibit a pronounced lack of perceived value in their work and experience a diminished sense of communal connectedness (Keyes, 1998; Rothmann, 2013). They tend to undervalue diversity, lack belief in others’ potential, and struggle to perceive coherent relationships within their workplace. The socially challenged profile demonstrated mixed results, characterized by inadequate social contribution and integration alongside low social actualization, acceptance, and coherence. Socially thriving individuals exhibited high functioning across all dimensions of social wellbeing. They reported feeling connected and valued within the organization, maintained an optimistic outlook on its potential, fostered strong relationships, and experienced a profound sense of belonging. The socially adequate profile demonstrated moderate functioning across all dimensions, indicating a satisfactory yet unremarkable level of social wellbeing. These individuals are neither struggling nor thriving, reflecting a balanced but not exceptional social wellbeing.

The level of social actualization was strikingly different between the socially thriving profile and the other profiles. Social actualization entails assessing the potential and trajectory of the organization and observing that the organization is developing, which creates hope and help employees to realize their social potential (Keyes, 1998; Rothmann, 2013). Therefore, individuals who are not socially actualized may perceive that they and others like them are not potential beneficiaries of social growth. The most notable difference between the socially disconnected profile and the other three social wellbeing profiles was regarding social contribution, i.e., individuals’ appraisals of their social value to their organization (Keyes, 1998; Rothmann, 2013) and mattering (Prilleltensky and Prilleltensky, 2021). Furthermore, two profiles (socially disconnected and socially challenged) functioned notably lower than the other two (socially thriving and socially adequate) regarding social coherence, which reflects their understanding and finding meaning in what is happening around them (Keyes, 1998; Colenberg et al., 2021). These findings align with a study by Moller and Rothmann (2019), which highlighted social actualization as an essential wellbeing component and identified social actualization and social coherence as the most problematic areas of social wellbeing for the managers in their study.

Significant differences were found between the four social wellbeing profiles regarding trust in managers and colleagues. This study showed that trust in managers and colleagues was influential in the promotion of employees’ social wellbeing and provided further support for previous research by Heyns and Rothmann (2021), Hennicks et al. (2022), and Kleynhans et al. (2022), who indicated positive associations between trust and employee flourishing at work. For instance, research findings of latent profile analyses of trust by Heyns and Rothmann (2021) demonstrated that high levels of trust in leaders stimulated confidence and the belief that one could achieve valued outcomes. In contrast, employees who did not trust their managers were less able to establish supportive relationships, were less engaged in their work, and were more inclined to leave the organization. Previous studies (Gambetta and Hamill, 2005; Edelman and Associates, 2009) have shown that social connectedness relies on trust. Our results showed that trust in managers matters significantly between the social wellbeing profiles. In line with the findings of Ren and Gray (2009), a lack of trust harms social wellbeing at work.

Job satisfaction and the intention to leave were strongly associated with social wellbeing. Individuals in the socially thriving profile (compared to the other profiles) experienced significantly higher job satisfaction and lower intentions to leave. These findings align with previous research that found strong positive associations between higher levels of social wellbeing and job satisfaction (Rothmann, 2013) and negative associations between higher levels of social wellbeing and intentions to quit (Redelinghuys et al., 2019). Social wellbeing facilitates interpersonal collaboration and reflects good social relationships (Cross et al., 2020). Individuals’ social relationships serve as a valuable resource (Hobfoll, 2001), reducing the likelihood of voluntary turnover (Ballinger et al., 2016).

Although members in the socially thriving and socially adequate profiles (representing 27.33% and 22.30% of the employees, respectively) had higher levels of trust in management and colleagues, social wellbeing, and job satisfaction, these profiles represented only 49.66%. This finding aligns with previous research, which indicated that less than half of the working population was not flourishing at work (Rothmann, 2013; Redelinghuys, 2016). In sum, although no previous research could be identified that rendered direct comparison of results possible, the current results provided further support for claims in the literature that individuals’ sense of wellbeing was related to how they perceived their social wellbeing (Keyes, 1998; Rothmann, 2013; Moller and Rothmann, 2019; Redelinghuys et al., 2019).

It is a matter of concern that younger employees and those with more years of service were more likely to be classified into the socially disconnected profile than the socially thriving profile. Younger employees, especially those with more experience, are critical for the public utility’s success. These findings imply that serious consideration should be given to the social wellbeing of younger employees. According to Keyes (2024), the age group from 20 to 40 is regarded as one of three life stretches where languishing is the highest. Job stressors and challenging life and work decisions in this age group result in stress and languishing. Critical for employees’ wellbeing, is the support from colleagues with whom they get along, who are there for them, and who create a collegial atmosphere (Keyes, 2024). Our finding about the association between trust in colleagues and social wellbeing confirms Keyes’ argument. By addressing the specific needs of each social wellbeing profile, organizations can foster a more positive, inclusive, and productive work environment, ultimately leading to better organizational outcomes.

By categorizing social wellbeing into these distinct profiles, our study offers a detailed perspective on how social contribution, social integration, social actualization, social coherence, and social acceptance interact within individuals. This level of detail might not have been apparent through a more generalized approach, thereby contributing to a sophisticated understanding of positive organizational psychology. Our findings advance the theoretical and practical understanding of social wellbeing in organizational settings, offering a foundation for future research and developing targeted interventions to improve employee wellbeing and organizational outcomes.

Organizations can foster a positive work climate conducive to social actualization by nurturing social connections and building trust in managers and colleagues. Managers can build trust by encouraging employees to share their work challenges, listening to them with genuine interest and empathy, removing stumbling blocks where possible, and emphasizing the meaningfulness of work (Seppälä and Cameron, 2015; VanderWeele, 2021). When workers have adequate social support structures and their suggestions are incorporated, they are more likely to feel socially accepted, that their social environment is coherent, and that they can actualize their potential. Consequently, they are more satisfied with their jobs and less likely to leave the organization (Tuzun and Kalemchi, 2012; Steffens et al., 2017). To further social acceptance and coherence, organizations can host events that create opportunities for social interaction and trust-building to empower colleagues’ camaraderie (VanderWeele, 2021).

## 6 Limitations and recommendations for future research

The study had various limitations that restricted the applicability of generalizations beyond the context of the sample subjects, industry, and country. For instance, a cross-sectional design was employed, which helped establish relationships – or their lack – among variables (Spector, 2019). However, this design could not test causality, and the results must be interpreted cautiously. Furthermore, the study sample only consisted of respondents employed by a significant role player (organization) in the utility industry; as a result, findings could not be generalized across different industries in South Africa. Self-report questionnaires were the best way to assess an individual’s experiences and perceptions (Spector, 2019). Still, at the same time, this method could not rule out the possibility of common method variance. Finally, this study considered a limited number of demographic variables; future studies might consider the inclusion of other correlates (e.g., ethnicity and gender). It might also be beneficial to compare the social wellbeing of employees who primarily work face-to-face with those who mainly work remotely or to consider the effects of short-term interactions versus long-term social relations.

## 7 Conclusion

Our findings showed that the study population was not a homogeneous group in terms of social wellbeing. Four distinct social wellbeing profiles were evident in a large South African utility organization: socially disconnected, socially challenged, socially thriving, and socially adequate. These profiles were closely linked to trust in managers and colleagues, with the social thriving profile showing significant differences from the other three profiles. The differing levels of social wellbeing among these profiles have substantial implications for job satisfaction and staff retention. This study provided a nuanced understanding of social wellbeing by identifying patterns in which social contribution, social integration, social actualization, social coherence, and social acceptance interacted within individuals in a population, which might otherwise not have been evident.

## Data availability statement

The datasets presented in this study can be found in online repositories. The names of the repository/repositories and accession number(s) can be found below: https://data.mendeley.com/datasets/xfrvh9tkwk/1.

## Ethics statement

The studies involving humans were approved by the Economic and Management Sciences Ethics Committee, North-West University. The studies were conducted in accordance with the local legislation and institutional requirements. The participants provided their written informed consent to participate in this study.

## Author contributions

EH took the lead in conceptualizing and writing the manuscript and collected and analyzed the data. MH and SR assisted with the data analyses, acted as an additional writer, and reviewed the manuscript. All authors contributed to the article and approved the submitted version.
